# When sleep daily rhythm meets the eye: sleep regularity as a behavioral signal of ocular aging

**DOI:** 10.1093/sleep/zsag165

**Published:** 2026-06-15

**Authors:** Kai-Yang Chen, Hoi-Chun Chan, Chi-Ming Chan

**Affiliations:** Department of General Medicine, Chang Gung Memorial Hospital (Linkou branch), Taoyuan, Taiwan; School of Pharmacy, China Medical University, Taichung, Taiwan; Department of Ophthalmology, Cardinal Tien Hospital, New Taipei City, Taiwan; Department of Ophthalmology, School of Medicine, Fu Jen Catholic University, New Taipei City, Taiwan

Age-related eye diseases are usually approached through familiar risk frameworks: genetics, vascular disease, metabolic dysfunction, intraocular pressure, oxidative stress, inflammation, and cumulative environmental exposure [1]. Sleep has increasingly entered this conversation, but most ophthalmic studies have focused on sleep duration, insomnia, snoring, or sleep-disordered breathing. These measures are clinically important, but they do not tell us whether sleep follows a regular daily rhythm. A person may sleep 7 hours nightly but maintain a highly unstable sleep–wake schedule. Sleep regularity, therefore, asks a different question: not only how much sleep is obtained, but how consistently sleep and wakefulness occur across days.

In this issue of *SLEEP*, Cui and colleagues extend this question to ocular aging in “Sleep Regularity and Age-Related Eye Diseases: Prospective Analyses with Retinal Imaging and Multimorbidity Assessment in the UK Biobank.” Using accelerometer-derived Sleep Regularity Index (SRI) data from 78 839 UK Biobank participants, the authors examined associations between sleep regularity and age-related macular degeneration (AMD), cataract, glaucoma, retinal vein occlusion, and diabetic retinopathy. They further linked SRI to retinal structural and vascular imaging markers and ocular multimorbidity. The SRI ranges from 0 to 100. A higher SRI means a more stable and regular sleep–wake pattern from day to day, while a lower SRI means more irregular sleep. In practical terms, higher SRI is the healthier direction, and lower SRI is the adverse direction. In the fully adjusted model of their study, each higher level of sleep regularity was associated with lower risk of AMD, cataract, and glaucoma, with hazard ratios (HRs) of 0.87, 0.95, and 0.89, respectively. The quintile analysis made the message even clearer. Compared with participants in the lowest SRI group, those in the highest SRI group had lower risk of AMD (HR 0.68), cataract (HR 0.87), and glaucoma (HR 0.75). In contrast, the associations with retinal vein occlusion and diabetic retinopathy were not consistent after full adjustment. Moreover, higher SRI was also associated with modestly greater macular, retinal ganglion cell, and retinal nerve fiber layer thicknesses, as well as more favorable retinal vascular geometry. Importantly, SRI declined with increasing ocular multimorbidity, suggesting that irregular sleep may track not merely with single disease entities but with a broader phenotype of ocular aging [2].

The novelty of this work lies less in proposing that sleep matters for eye health, which is an increasingly accepted concept, than in shifting attention toward sleep regularity as a circadian dimension of sleep health. The SRI captures the probability of being in the same sleep–wake state at the same clock time on consecutive days, integrating variability in sleep onset, wake time, nocturnal fragmentation, and multi-segment sleep [3, 4]. This differs meaningfully from sleep duration. Large cohort studies have linked irregular sleep timing to mortality, major cardiovascular events, dementia risk, and brain structural differences, often independent of total sleep duration [5–7]. Cui et al. now suggest that this principle may extend to the retina, lens, and optic nerve.

Biological plausibility is strong. The retina is not merely a passive photoreceptive tissue; it is a circadian-regulated neural structure with intrinsic clocks in photoreceptors, retinal pigment epithelium (RPE), retinal ganglion cells, Müller glial cells, and vascular cells [8–10]. Daily rhythmicity influences photoreceptor outer-segment shedding, dopamine and melatonin signaling, visual sensitivity, vascular tone, and metabolic homeostasis. Circadian disruption could plausibly impair RPE–photoreceptor interactions, increase oxidative stress, alter choroidal and retinal perfusion, promote inflammatory signaling, and destabilize neurovascular coupling. These mechanisms resonate with AMD, in which mitochondrial dysfunction, RPE stress, impaired quality control, and chronic inflammation are increasingly recognized as central biological processes [11, 12]. Müller glial cells may also be relevant because they participate in metabolic support, extracellular homeostasis, inflammatory regulation, and neuroprotection in retinal aging and AMD [13].

Inflammatory mechanisms provide another bridge between sleep regularity and ocular aging. Irregular sleep has been associated with cardiometabolic stress, inflammatory activation, and endothelial dysfunction, processes that may affect the retina and optic nerve because of their high metabolic demand and dense microvascular dependence [14]. Purinergic inflammatory signaling, including P2X7 receptor-related pathways, has also been implicated in ocular inflammatory and degenerative disease mechanisms, offering one possible molecular link between metabolic stress, innate immune activation, and retinal neurodegeneration [15]. Cataract may similarly relate to sleep irregularity through oxidative stress, systemic inflammation, metabolic dysregulation, and diabetes-related lens vulnerability [16, 17].

The glaucoma finding is particularly interesting. Glaucoma is not only a disease of intraocular pressure. Vascular regulation, optic nerve perfusion, autonomic tone, and retinal ganglion cell vulnerability are also important, especially in normal-tension glaucoma [18, 19]. Retinal ganglion cells are metabolically demanding neurons, and chronic instability in sleep–wake timing may amplify vascular dysregulation, oxidative stress, and neuroinflammatory susceptibility. This is particularly relevant for glaucoma phenotypes in which perfusion and hemorheological factors may contribute alongside intraocular pressure [20]. Also, sleep irregularity could plausibly affect nocturnal blood pressure, intraocular pressure rhythm, ocular perfusion pressure, and inflammatory stress. This does not prove causality, but it provides a reasonable framework for future mechanistic studies.

The imaging component is especially valuable. Although the observed retinal structural and vascular correlations were modest, their directionality provides an anatomical bridge between behavioral sleep metrics and ocular outcomes. Greater retinal ganglion cell and retinal nerve fiber layer thickness among more regular sleepers are compatible with a neuroretinal resilience signal, while more favorable vascular geometry may reflect healthier systemic and ocular microvascular regulation. These findings should not be interpreted as diagnostic biomarkers at the individual level, but they enrich the epidemiological association by anchoring it to measurable retinal phenotypes. The relevance of retinal vascular signals is supported by evidence that retinal microvascular features reflect systemic vascular and neurological health [21, 22].

Several aspects deserve emphasis. First, sleep regularity was measured objectively with a wearable device rather than by questionnaire, allowing a more accurate assessment of day-to-day sleep timing. Second, the authors examined multiple eye diseases rather than a single outcome, allowing them to distinguish stronger associations for AMD, cataract, and glaucoma from weaker or absent associations for retinal vein occlusion and diabetic retinopathy. Third, the analysis incorporated dose–response modeling, subgroup analyses, propensity-score matching, and prediction modeling. Subgroup analyses showed the same protective direction across age, sex, smoking, alcohol consumption, and hypertension strata. Propensity-score matching also supported the main pattern, with lower-SRI participants showing higher post-matching prevalence of AMD, cataract, and glaucoma than higher-SRI participants: 3.3 per cent versus 2.0 per cent, 8.5 per cent versus 6.8 per cent, and 2.8 per cent versus 1.6 per cent, respectively. Prediction models incorporating SRI achieved areas under the curve (AUCs) of 0.734 for AMD, 0.720 for cataract, and 0.723 for glaucoma in the test sets. Fourth, the multimorbidity analysis is clinically meaningful. Older patients commonly present not with isolated ocular disease but with overlapping lens, retinal, optic nerve, and systemic vascular pathology. A sleep metric that scales with multimorbidity may serve as a practical marker of global ocular vulnerability. The principal clinical interpretation of these findings is summarized in Table 1.

**Table 1:** Clinical Interpretation of the Cui et al. Findings

Finding	Interpretation	Caution
Lower SRI associated with higher AMD, cataract, and glaucoma risk	Sleep regularity may mark ocular aging vulnerability	Observational association, not proof of causality
No robust association with retinal vein occlusion or diabetic retinopathy	Effects may be disease-specific rather than universal	Outcome ascertainment and confounding remain relevant
Higher SRI associated with modestly healthier retinal structure and vascular geometry	Supports biological plausibility through neuroretinal and microvascular correlates	Effect sizes may not be individually diagnostic
SRI declines with ocular multimorbidity	Sleep irregularity may reflect the global burden of ocular aging	Multimorbidity patterns require external validation

Abbreviations: AMD, age-related macular degeneration; SRI, Sleep Regularity Index.

Nevertheless, the translational message should remain cautious. This is an observational study, and residual confounding cannot be excluded. Sleep regularity is socially and biologically patterned: it varies with occupation, income, education, physical activity, mental health, cardiometabolic status, medication use, caregiving responsibilities, and environmental light exposure. Even extensive adjustment cannot fully remove these influences. Accelerometry was measured over approximately 1 week, which may not capture long-term habitual sleep regularity. Retinal imaging and accelerometry were obtained during different UK Biobank assessment phases, limiting temporal inference for imaging correlations. Disease ascertainment through linked health records may under-detect milder ophthalmic disease. Finally, UK Biobank participants are generally healthier and less socioeconomically diverse than the general population, which may affect generalizability.

The clinical question, therefore, is not whether SRI should immediately become an ophthalmic screening test. Rather, the more useful question is whether sleep regularity should be incorporated into the expanding concept of eye-healthy behavior. Ophthalmologists already counsel patients regarding smoking cessation, glycemic control, blood pressure, diet, ultraviolet protection, and disease-specific surveillance. Sleep regularity may eventually join this list, not as a replacement for established risk factors, but as a low-cost behavioral signal reflecting circadian stability and systemic health. For sleep clinicians, the study reminds us that the consequences of irregular sleep may be visible in the eye, an accessible extension of the central nervous system and microvasculature.

Future studies should address causality and intervention. Repeated accelerometry over longer periods could clarify whether persistent sleep irregularity predicts incident ocular disease more strongly than a single measurement window. Mendelian randomization, negative-control analyses, and within-person longitudinal imaging could help separate causal pathways from confounding. Randomized interventions that stabilize sleep timing, optimize morning light exposure, reduce nocturnal light exposure, or improve circadian alignment may test whether modifying sleep regularity alters retinal structural trajectories or ocular disease risk. Such studies should also examine whether effects differ across AMD phenotypes, glaucoma subtypes, diabetic status, and shift-work exposure.

Cui et al. have opened a timely and provocative line of inquiry. Their findings show that higher SRI, reflecting more regular sleep, is associated with lower risks of AMD, cataract, and glaucoma, although the study does not establish causality. Nevertheless, their work supports a broader view of ocular aging, one in which sleep timing deserves consideration alongside vascular, metabolic, inflammatory, and genetic risk factors. The retina records far more than images; it may also reflect the rhythm of daily life. In that sense, sleep regularity may become an important behavioral window into the aging eye.

## Disclosure statement

*Financial disclosure*: The authors report no financial arrangements or connections related to this work.

*Non-financial disclosure*: The authors report no potential non-financial conflicts of interest related to this work.

## References

[1] Fleckenstein M, Keenan TDL, Guymer RH, et al. Age-related macular degeneration.. *Nat Rev Dis Primers*. 2021;7(1):31. 10.1038/s41572-021-00265-2 PMC12878645 33958600

[2] Cui X, Yuan J, Zhao Q, Yu-Wai-Man P, Zheng T. Sleep regularity and age-related eye diseases: prospective analyses with retinal imaging and multimorbidity assessment in the UK Biobank. *Sleep*. 2026. 10.1093/sleep/zsag111 42017838

[3] Lunsford-Avery JR, Engelhard MM, Navar AM, Kollins SH. Validation of the sleep regularity index in older adults and associations with cardiometabolic risk. *Sci Rep*. 2018;8(1):14158. 10.1038/s41598-018-32402-5 30242174 PMC6154967

[4] Fischer D, Klerman EB, Phillips AJK. Measuring sleep regularity: theoretical properties and practical usage of existing metrics. *Sleep*. 2021;44(10):zsab103. 10.1093/sleep/zsab103 PMC8503839 33864369

[5] Cribb L, Sha R, Yiallourou S, et al. Sleep regularity and mortality: a prospective analysis in the UK Biobank. *Elife*. 2023;12:RP88359. 10.7554/eLife.88359 PMC10666928 37995126

[6] Chaput JP, Biswas RK, Ahmadi M, et al. Sleep regularity and major adverse cardiovascular events: a device-based prospective study in 72 269 UK adults. *J Epidemiol Community Health*. 2025;79(4):257–264. 10.1136/jech-2024-222795 39603689 PMC12066246

[7] Yiallourou SR, Cribb L, Cavuoto MG, et al. Association of the Sleep Regularity Index with incident dementia and brain volume. *Neurology*. 2024;102(2):e208029. 10.1212/wnl.0000000000208029 38165323 PMC13528887

[8] Ko GY . Circadian regulation in the retina: from molecules to network. *Eur J Neurosci*. 2020;51(1):194–216. 10.1111/ejn.14185 30270466 PMC6441387

[9] Meyer N, Harvey AG, Lockley SW, Dijk DJ. Circadian rhythms and disorders of the timing of sleep. *Lancet*. 2022;400(10357):1061–1078. 10.1016/s0140-6736(22)00877-7 36115370

[10] Zeng Y, Rong R, You M, Zhu P, Zhang J, Xia X. Light-eye-body axis: exploring the network from retinal illumination to systemic regulation. *Theranostics*. 2025;15(4):1496–1523. 10.7150/thno.106589 39816683 PMC11729557

[11] Shu DY, Chaudhary S, Cho KS, et al. Role of oxidative stress in ocular diseases: a balancing act. *Metabolites*. 2023;13(2):187. 10.3390/metabo13020187 PMC9960073 36837806

[12] Chen KY, Chan HC, Lin WW, Chan CM. Mitochondrial dynamics and their role in the pathogenesis of age-related macular degeneration: a comprehensive review. *Redox Biol*. 2025;93:103976. 10.1016/j.redox.2025.103976 41935442 PMC13089171

[13] Chen KY, Chan HC, Hwang YS, Lin WW, Chan CM. Are Müller glial cells gatekeepers of neuroprotection and regeneration in age-related macular degeneration? Unraveling their roles in pathophysiology and therapeutic innovation. *Prog Retin Eye Res*. 2026;112:101471. 10.1016/j.preteyeres.2026.101471 41985834

[14] Zhang C, Qin G. Irregular sleep and cardiometabolic risk: clinical evidence and mechanisms. *Front Cardiovasc Med*. 2023;10:1059257. 10.3389/fcvm.2023.1059257 36873401 PMC9981680

[15] Chen KY, Chan HC, Chan CM. Unveiling the P2X7 receptor: exploring its mechanisms, pathogenic role in ocular diseases, and emerging therapeutic potential. *Mol Aspects Med*. 2025;105:101389. 10.1016/j.mam.2025.101389 40729957

[16] Chen KY, Chan HC, Chan CM. How does diabetes shape the landscape of cataract development and surgical success? A systematic review and meta-analysis. *Endocrine*. 2025;90(2):404–419. 10.1007/s12020-025-04374-w 40775566

[17] Cvekl A, Vijg J. Aging of the eye: lessons from cataracts and age-related macular degeneration. *Ageing Res Rev*. 2024;99:102407. 10.1016/j.arr.2024.102407 38977082 PMC11288402

[18] Ikegami K . Circadian rhythm of intraocular pressure. *J Physiol Sci*. 2024;74(1):14. 10.1186/s12576-024-00905-8 38431563 PMC10908160

[19] Mursch-Edlmayr AS, Bolz M, Strohmaier C. Vascular aspects in glaucoma: from pathogenesis to therapeutic approaches. *Int J Mol Sci*. 2021;22(9):4662. 10.3390/ijms22094662 PMC8124477 33925045

[20] Cheng HC, Chan CM, Yeh SI, Yu JH, Liu DZ. The hemorheological mechanisms in normal tension glaucoma. *Curr Eye Res*. 2011; 36(7):647–653. 10.3109/02713683.2010.521876 21609272

[21] Cheung CY, Ikram MK, Sabanayagam C, Wong TY. Retinal microvasculature as a model to study the manifestations of hypertension. *Hypertension*. 2012;60(5):1094–1103. 10.1161/hypertensionaha.111.189142 23045470

[22] London A, Benhar I, Schwartz M. The retina as a window to the brain—from eye research to CNS disorders. *Nat Rev Neurol*. 2013;9(1):44–53. 10.1038/nrneurol.2012.227 23165340

